# *On the Road to Recovery* psychological therapy versus treatment as usual for forensic mental health patients: study protocol for a randomized controlled feasibility trial

**DOI:** 10.1186/s40814-018-0319-z

**Published:** 2018-07-13

**Authors:** Lindsey G. McIntosh, Morag Slesser, Suzanne O’Rourke, Lindsay D. G. Thomson

**Affiliations:** 1 Division of Psychiatry, University of Edinburgh, Edinburgh, EH14 5HF UK; 2 The State Hospitals Board for Scotland, Carstairs, ML11 8RP UK; 3 Department of Clinical Psychology, University of Edinburgh, Edinburgh, EH8 9AG UK

**Keywords:** Mentally disordered offenders, Feasibility outcomes, RCT, Recovery, Coping ability, Clinical insight, Psychological intervention

## Abstract

**Background:**

*On the Road to Recovery* (OTRTR) is a brief low intensity group psychological therapy that aims to improve patients’ insight into their mental disorder and develop adaptive coping skills to help manage distress. OTRTR is currently delivered in forensic mental health services in Scotland. However, to date, this therapy has not been evaluated as to its effectiveness or safety for forensic patients.

**Methods:**

This is a parallel-group feasibility randomized controlled trial with single-blind assessments comparing OTRTR therapy to treatment as usual (TAU) for forensic mental health patients. Fifty participants will be recruited from high, medium, and low secure forensic mental health services in Scotland. Participants will receive OTRTR for approximately 12 weeks or continue treatment as usual for 12 weeks. The trial’s primary aims relate to testing the acceptability and feasibility of key trial procedures that would be necessary for a definitive RCT of OTRTR. The secondary aims include estimating therapeutic effect sizes on clinical outcomes including insight and coping skills. The study design also features an adverse event monitoring plan to estimate the safety of OTRTR for participants, as well as use of intensive longitudinal methods to identify “key ingredients” to the OTRTR therapy protocol.

**Discussion:**

This study will inform the design and sample size for a future full-scale randomized controlled trial (RCT), which will be conducted to determine the effectiveness of the *On the Road to Recovery* intervention in improving forensic mental health patients’ clinical insight and coping skills.

**Trial registration:**

ISRCTN Registry, ISRCTN75126867 registered 27 July 2017

## Background

Forensic mental health patients often present with varied and complex treatment needs relating to their mental health and offending behavior [1, 2]. Treating these individuals requires significant involvement of clinical and forensic psychological practitioners as part of the multi-disciplinary treatment team. However, the evidence base for psychological interventions with forensic patients remains severely limited. Previous reviews have highlighted that a lack of rigorous methodologies, including the randomized controlled design, in forensic mental health evaluations has restricted the synthesis of effectiveness evidence in this field [2, 3].

Within NHS Scotland, psychological treatment of forensic patients is guided by the Forensic Matrix (an addendum to the larger “Matrix,” a guide to delivering evidence-based psychological therapies in Scotland [4]). The Forensic Matrix proposed a model of “stepped care” [5] for forensic patients. This model acknowledges that forensic patients often present with complex and enduring problems relating to their risk and offending behaviors which require highly specialist and individually tailored psychological treatment from practitioners with high levels of training and forensic expertise. However, they often also have simpler underlying or associated psychological needs which may respond to less intensive interventions. According to the Forensic Matrix stepped care approach, different levels of intervention intensity can be used to address forensic patients’ needs. For example, a forensic patient may require psychological work aimed at improving their management of basic emotions (low intensity) before the patient will be able to cope with the demands of sex-offending treatment (specialist/highly specialist). Several psychological interventions which address different common underlying needs of forensic patients have now been developed as part of the Forensic Matrix suite of interventions. There is now a critical need to evaluate the effectiveness of these therapies.

*On the Road to Recovery* (OTRTR) is a brief, low intensity group psychological intervention delivered across the Scottish Forensic Mental Health Managed Care Network (“Forensic Network”). The OTRTR protocol was written in 2011 by a working group of clinical and forensic psychologists within the Forensic Network. The program is based on the principles of cognitive behavior therapy with a focus on compassionate mind training [6]. The core purpose of OTRTR is to open a dialog about recovery and instill in patients hope that recovery is possible. It is intended to be one of the first psychological treatments offered to patients on their journey to recovery from mental health difficulties, prior to engaging in more demanding and longer term therapy.

The aim of this study is to determine the feasibility of conducting an RCT comparing *On the Road to Recovery* psychological therapy to treatment as usual (TAU) for forensic mental health patients. Consistent with the UK Medical Research Council guidance (MRC) results from this study will directly inform the decision to undertake a future definitive RCT of OTRTR [4, 5]. Specifically, the feasibility RCT will determine:

1. Participant recruitment, consent, and retention rates
2. Acceptability of randomization to treatment condition
3. Acceptability of the OTRTR intervention for patients
4. Safety of the OTRTR intervention for patients
5. Acceptability of intensive longitudinal modeling procedures, in terms of:
  - The completion rate of standardized recording forms of the therapy content delivered by OTRTR therapy facilitators
  - The completion rate of primary clinical outcome measures weekly during treatment phase
6. Obtain estimates of likely therapeutic effects of OTRTR for use in sample size calculation of a future definitive trial. The between-group mean difference in therapeutic outcomes at the post treatment assessment, along with the standard deviation of the outcome for the entire sample, at baseline will be recorded.

## Methods

### Design

This study is a feasibility study using a parallel-group randomized outcome blinded design to compare OTRTR to treatment as usual (TAU). As a feasibility study, the primary study aims relate to testing the acceptability and feasibility of key trial procedures that would be necessary for a full-scale RCT of this program. The comparator group will receive treatment as usual. In this study, 50% of participants will be randomized to commence OTRTR immediately for approximately 12 weeks. The remaining 50% will engage in TAU for 12 weeks and may complete OTRTR in the future. Participants will continue their usual access to concomitant therapies and therapeutic activities throughout the study. Participants will complete a range of self-report measures and a clinical interview at baseline (T0), post treatment (T1), and at follow-up (T2), 3-months after the T1 assessment, with an assessor who will be blind to participants’ allocation status. Clinical effect sizes will be estimated using two approaches, at the group level for a range of clinical outcomes by comparing change from pre to post treatment and at the individual level for the two primary outcomes—insight into mental disorder and use of adaptive coping skills—using intensive longitudinal methods (ILM) [7, 8]. Findings from two large treatment evaluations, the PEPS trial [9] and Ministry of Justice’s evaluation of the prison-based Core Sex Offender Treatment Programme [10], demonstrated that psychological therapies have the potential to cause harm to patients, a concept which has received little attention until recently. Accordingly, this study will pilot an adverse event monitoring plan to estimate the safety of OTRTR for participants.

Following participation in the study, participants will be offered the opportunity to complete a semi-structured interview which will gather qualitative information on their experiences of participating and views on different aspects of the trial (for instance, randomization, treatment, and assessments).

### Study setting

Participants will be recruited and identified from forensic mental health services which are part of the NHS Scotland. Services include high, medium, and low secure inpatient settings as well as community forensic teams.

### Sample size

As this study is a feasibility trial, a power calculation to determine sample size was not undertaken [11]. The target recruitment for this feasibility trial will be *N* = 50 (25 in each intervention arm). This figure is consistent with recommendations for pilot and feasibility studies and appropriate given the study aims [12, 13].

### Inclusion criteria

People meeting inclusion criteria for the study include (1) males and females aged between 18 to 65 years, (2) who are receiving treatment under the Mental Health (Care and Treatment) (Scotland) Act 2003 from a participating forensic mental health service, and (3) who are viewed by their Responsible Medical Officer (RMO) as capable of providing informed consent and well enough to participate in the study.

### Exclusion criteria

People meeting exclusion criteria for the study include (1) those with a diagnosis of intellectual disability, (2) those who are viewed by their RMO as incapable of providing consent or are too unwell to participate, and (3) those who have completed *On the Road to Recovery* in the prior 3 years.

### Identification and recruitment of participants

The study inclusion criteria were selected to match the broad range who are referred to On the Road to Recovery. Potential participants will be identified by their local psychological therapies team using the existing local referral process. The Responsible Medical Officers for each patient referred to On the Road to Recovery will be consulted on whether their patient has capacity to provide informed consent. Those without capacity will not be approached about the research. Potential participants with capacity to consent will be first informed of the study by their local psychological therapies team. Subsequently, a researcher will meet with these potential participants that have expressed an interest to provide them with further information and obtained informed consent to participate.

### Randomization

#### Sequence generation and allocation

Participants will be randomly assigned to either OTRTR or TAU with a 1:1 allocation ratio and varying block size (4 or 6), using a computer-generated randomization schedule stratified by site and assessed treatment need at T0. Participants will be categorized as having *low* or *high* treatment need based on scores on the two primary clinical outcome measures: the Birchwood Insight Scale and the Coping Styles Questionnaire. To categorize participants, we will employ cut-off scores of ≤ 9 (the cut-off for good versus poor insight following Birchwood et al. [14]) on the Birchwood Insight Scale and ≤ 37 on the Coping Styles Questionnaire [15] adaptive coping index (corresponding to 1 SD below the observed sample mean for State Hospital patients commencing *On the Road to Recovery* in a previous evaluation (McIntosh, Purcell, O’Rourke, Slesser, Thomson, manuscript in preparation)). Participants with poor insight and low adaptive coping skills will be considered to have *high* treatment need, and all other participants will be considered to have *low* treatment need. This stratum will be used to equally distribute patients with more acute needs across the two treatment conditions.

#### Implementation

Staff of the Forensic Network office will generate the allocation sequence using an online randomization service (www.sealedenvelope.com). The Forensic Network office provides a centralized staff group who are knowledgeable of the participating services (the potential participating sites are part of the “Forensic Network”) but also independent from the trial research team. The Forensic Network office will generate the allocation sequences using an online randomization service (www.sealedenvelope.com). The researcher completing the baseline assessment will communicate the information necessary for allocation (site and assessed treatment need) to the Forensic Network office within 3 days of the participants’ T0 assessment. The Forensic Network office will allocate the participant and relay this information to the local investigators by email and keep a randomization record to audit compliance with the randomization procedure.

### Blinding

The assessor administering measures at T0, T1, and T2 assessments will be blind to participants’ allocation status. Participants and OTRTR facilitators will not be blind to allocation status. Participants will be asked not to divulge information about their group to the assessor. Any instances of unblinding will be recorded, and the clinical outcome analysis will be repeated excluding these participants to determine the robustness of the findings.

### Description of the intervention

#### On the Road to Recovery

The OTRTR program is comprised of two brief treatment modules: *Awareness & Recovery* (A&R) and *Looking After Yourself* (LAY). A&R provides psychoeducational material adapted from Morrison et al. [16] “Think you’re crazy? Think Again” and from “Coping with Mental Illness” [17], the original psychoeducation program delivered across the Forensic Network. The LAY program’s core aim is to help patients develop basic coping skills to manage psychological distress and to develop a “toolkit” for coping with this distress now and in the future. The protocol is semi-structured and organized by topics instead of sessions, which allows the program to flexibly match pace to groups with greater or lesser need by adjusting the number of sessions. Each topic contains several objectives that are accompanied by structured didactic activities, exercises, and discussion prompts. The program will run over approximately 12 weeks, with weekly 2-h sessions. Treatment delivery is primarily in small groups; however, the program can also be delivered on an individual basis as part of the study. Participant attendance and engagement in the treatment sessions will be monitored and recorded. Participants’ RMOs may withdraw a participant from the study and therefore discontinue OTRTR sessions should there be a deterioration in participants’ clinical presentation.

OTRTR therapy facilitators will receive group supervision at least once every four sessions delivered, consistent with the Forensic Matrix governance standards [18]. Facilitators will be provided with standardized recording documents to closely record the program content delivered in each session.

#### Treatment as usual

Participants randomized to the TAU condition will engage in treatment as usual including access to other psychological therapies, with the exception that they do not attend OTRTR sessions. TAU participants will be advised they will be offered the OTRTR therapy at a later stage. Access to and engagement in non-psychological therapies differs by participating site and individual participants. For both groups, we will record the care received by participants during the trial to enable a full description of treatment as usual.

### Safety monitoring

We will monitor potential harm from study treatments experienced by participants by recording the occurrence of adverse (AE) and serious adverse events (SAE). Any AE and SAE experienced by participants after consenting to the study will be recorded and reported regardless of whether it is likely that they are the result of treatment received. The Health Research Authority defines SAE as an occurrence that (a) results in death, (b) is life-threatening, (c) requires hospitalization or prolongation of existing hospitalization, (d) results in persistent or significant disability or incapacity, and (e) consists of a congenital anomaly or birth defect. Applying this definition to psychiatric inpatient settings, acts of serious self-harm, attempted suicide, and suicide would qualify as a SAE. Events that constitute SAEs are already routinely collected and monitored at all participating sites using the Datix incident reporting software.

AEs will be similarly monitored throughout the study. In the present study, an AE is considered to have occurred if either of the following takes place: (a) participant is removed from the study at the request of their RMO due to significant deterioration in the patient’s mental state and/or behavior and (b) a participant’s global CORE-OM score (indexes overall psychiatric distress) increases from the previous assessment to an extent that is both clinically significant and reliable (as defined in Evans et al. [19]). All SAEs and AEs will be recorded, and SAEs will be reported to a NHS Scotland Research Ethics Committee if and when they occur.

### Data collection and outcome measures

#### Participant timeline

**Fig. 1:**
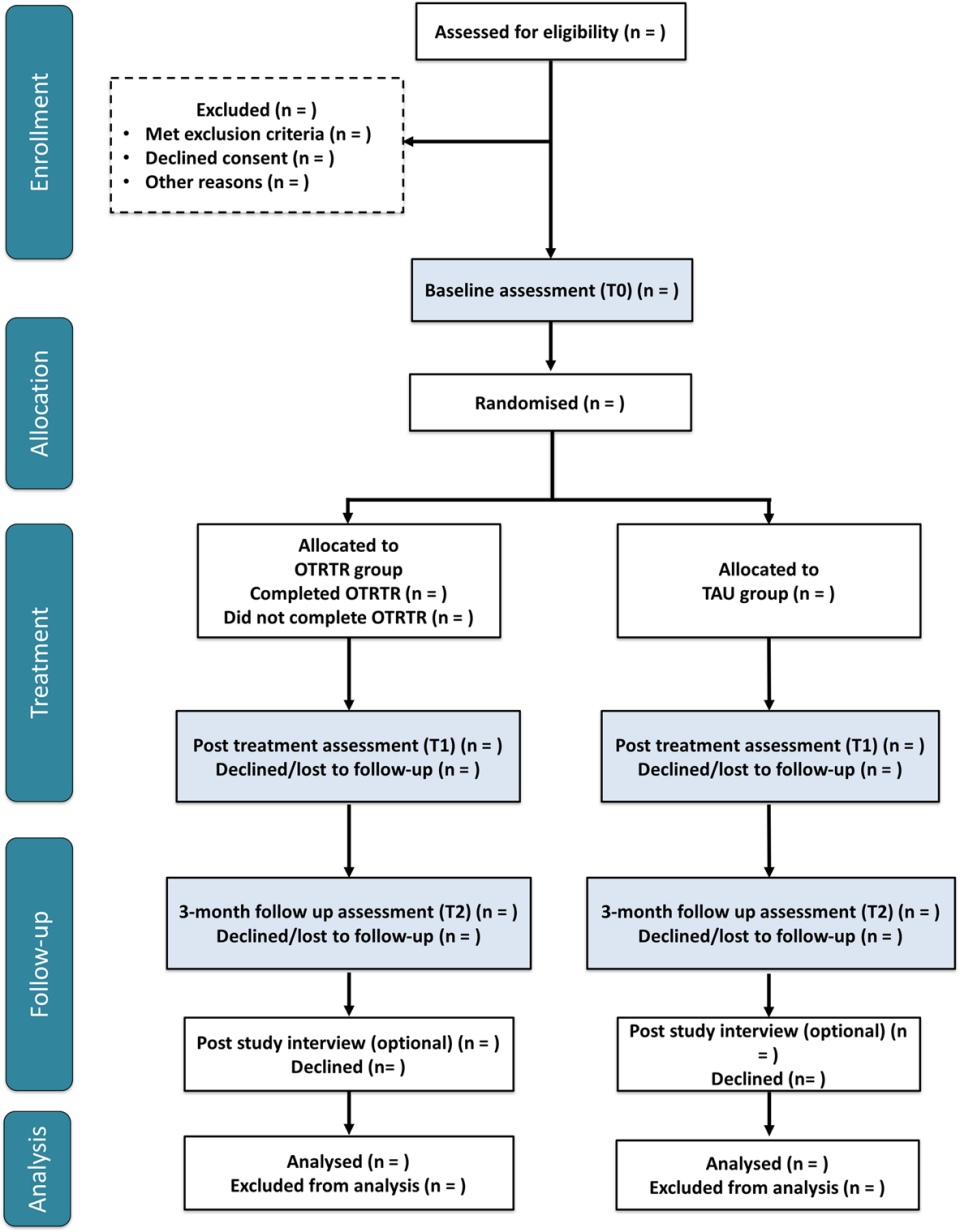
Participant flow diagram

The trial consists of a 12-week treatment phase with a 3-month (13-week) follow-up phase. As shown in Fig. 1, participants in both treatment conditions will be assessed pretreatment at T0, post treatment at T1, and 3-months after post-treatment at T2. A schedule of study procedures and assessments is detailed in Table 1.

**Table 1:** Schedule of study procedures and assessments

	Study period						
	Recruitment	Baseline (T0)	Allocation	Treatment phase	Post treatment (T1)	3-month follow-up (T2)	Post-study Interview (optional)
Procedures							
Eligibility screen	X						
Assess capacity	X						
Informed consent	X						
Randomization			X				
Assessments							
Birchwood Insight Scale (BIS)		X		X (weekly)	X	X	
Coping Styles Questionnaire (CSQ)		X		X (weekly)	X	X	
Clinical Outcomes in Routine Evaluation (CORE-OM)		X			X	X	
Rosenberg Self-Esteem Scale (RSE)		X			X	X	
Questionnaire on the Process of Recovery (QPR)		X			X	X	
Brief Psychiatric Rating Scale (BPRS)		X			X	X	
Letter-Number Sequencing Test (LNST)		X			X	X	
Study entrance questionnaire		X					
Post-study interview							X
Intervention							
On the Road to Recovery				X			
Treatment as usual				X			

#### Baseline measures

The following measures will be collected at T0. They will be used as moderator variables in analysis to identify mechanisms of change and predict therapy outcomes at the individual level. We will explore the associations between these variables and change on the clinical outcome measures from T0 to T1.

- Demographic data (age, sex, ethnicity, education level)
- Primary and secondary psychiatric diagnoses
- Medication at each assessment point
- Previous participation in psychological therapies
- Time since admission (months)
- *Verbal working memory*: The Letter-Number Sequencing subtest of the WAIS-IV [20] will be used to index verbal working memory. Working memory was identified as one of several cognitive abilities which may impact patients’ ability to engage with and benefit from the On the Road to Recovery intervention.

#### Primary outcome measures

Primary study outcomes relate to the feasibility and acceptability of key trial procedures.

1. Number of eligible participants identified over the study period will be indexed by the total number of participants identified across sites who meet eligibility criteria.
2. Estimate rate of recruitment into the trial
  - The proportion of eligible patients who agree to participate (provide consent): We anticipate the consent refusal rate to fall in the range of 16–37% based on findings from previous RCTs of psychological interventions in forensic populations [21–24]. The adopted benchmark of success in this study is a consent refusal rate in this study of less than 37%.
  - The number of participants enrolled into the study each month during the recruitment period: As in other ongoing feasibility RCTs [25], the benchmark of recruitment success is achieving 80% of target sample size, in this case recruiting at least 40 participants into the study.
3. Audit adherence to randomization procedure: The number of instances where the actual treatment allocation differed from assigned allocation. Instances where the participant is removed from the study by his or her RMO, who cites a reason related to the allocated condition as opposed to deterioration in clinical presentation, will also be considered non-adherence to the randomization process. Any instances of non-adherence to the randomization procedure will cause great concern as to the feasibility of a future phase III RCT of OTRTR.
4. Estimate completion rate of OTRTR: Proportion of participants who complete OTRTR. Following similar studies [26, 27] participants will be considered to have completed OTRTR if they attended at least 80% of the planned sessions.
5. Explore reasons for study drop-out during the study treatment: Participants will have the option of providing a reason they do not wish to continue with the study before exiting the study.
6. Completion rate of primary clinical outcome measures weekly during treatment phase: The proportion of Birchwood Insight Scale (BIS) and Coping Styles Questionnaire (CSQ) forms completed as intended each week during the treatment phase will be calculated.
7. Completion rate of standardized recording forms of the therapy content delivered by OTRTR therapy facilitators: Proportion of complete forms will be calculated.
8. Number of participants lost to follow-up and reasons: Number of participants who withdrew from the study or who were otherwise lost to follow-up, with reasons recorded (if provided). The adopted benchmark of success is a participant attrition rate of less than 20% at the post-treatment (T1) assessment.
9. Safety of OTRTR for patients: Descriptive statistics for frequency of observed AEs and SAEs across treatment conditions. Adverse events may occur for reasons independent of the therapy being studied due to the nature of this clinical population. Safety of OTRTR will be assessed by comparing the rates of SAEs and AEs across the two study treatment conditions. We will also compare the incident rate of SAEs for study participants to the rate observed for each participant in the 12 weeks prior to enrolment in the study. This reference data will be requested from the local site clinical effectiveness departments or equivalents following study completion.
10. Average duration of assessment battery at T0, T1, and T2. Descriptive statistics for length of assessment. We anticipate the T0 assessment will last approximately 90 min, and the T1 and T2 assessments 60 min each.

#### Semi-structured interview

After T2 assessment, each participant will be offered the opportunity to complete a semi-structured interview with the researcher. Interviews will solicit participants’ experiences of participating in the study, with a focus on the treatment phase and assessments. These results will inform the design of a future RCT of OTRTR.

#### Secondary outcomes

The secondary outcome measures relate to intended treatment outcomes of OTRTR. The following measures will be completed at baseline (T0), post treatment (T1), and 3-month follow-up (T2) by a researcher blind to the participant’s allocation status.

1. Birchwood Insight Scale (BIS) [14] is an eight-item self-report measure of insight. The original study established BIS total score test-retest reliability (1-week) as *r* = 0.90 and internal consistency of *α* = 0.75. The BIS variable used in this study will be the total score (max 12).
2. Coping Styles Questionnaire (CSQ) [15] is a 60-item self-report measure on the use of maladaptive and adaptive coping styles. It is comprised of four subscales, each a unique dimension of coping: rational, detached, emotional, and avoidance. Test-retest reliability of the CSQ subscales range from *r =* 0.70 to 0.80, and the subscale internal consistencies of *α* = 0.69 to 0.90. The CSQ variable used in this study will be the total of the two adaptive coping subscales, Rational and Detached.
3. Clinical Outcomes in Routine Evaluation Outcome Measure (*CORE-OM*) [28] is a 34-item self-report measure of psychological distress that covers four domains: wellbeing, symptoms/problems, functioning, and risk to self and others. A global measure of distress is also calculated across these domains. Good psychometric properties for this measure in non-clinical, clinical, and forensic populations have been established [19, 29]. The CORE-OM variable used in this study will be the 34-item mean score (max 4).
4. Rosenberg Self-Esteem Scale (RSE) [30] is a 10-item self-report measure of self-esteem. The RSE has an internal consistency of *α* = 0.77 and two-week test-retest reliability of *r* = 0.85. The total RSE score will be used (max 30).
5. Questionnaire on the Process of Recovery (QPR) [31] is a 22-item measure of service user-rated recovery comprised of an interpersonal and intrapersonal subscale. The initial validation study found acceptable two-week test-retest reliability (intrapersonal subscale *r* = 0.87, interpersonal subscale *r* = 0.77) and internal consistency (intrapersonal *α* = 0.94, interpersonal *α* = 0.77). The intrapersonal (max 68) and interpersonal (max 20) subscale scores will be used.
6. Brief Psychiatric Rating Scale (BPRS) [32] is a clinician/researcher-rated tool used to assess severity of psychopathology. The 18 items sum to derive a continuous total score of symptom severity. The BPRS has good psychometric properties is the most widely used outcome measure in treatment studies across populations with severe and persistent mental illness [33]. The total BPRS score will be used (max 126).
7. Institution-recorded incidents of physical aggression and violence: The number of incidents of violence/aggression, for which the participants are considered to have responsibility in instigating or exacerbating, during the study treatment phase and 3-month follow-up period will be compared across treatment groups. This information will be as participants complete T2.
8. Institutional privileges: Changes in institutional privileges (e.g., increased grounds access, unsupervised phone calls, and patient outings) will be recorded for all participants during the study treatment phase and 3-month follow-up period. This information will be after participants complete T2.

#### Weekly clinical measures

Following an intensive longitudinal methods (ILM) approach [7, 8], the BIS and CSQ will be administered to all participants each week during the 12-week study treatment phase. Members of the local health care team will administer these weekly measures and as such they will not be blind to participants’ treatment allocation status.

### Participant and data retention

Participants may withdraw from the study at any time. We will offer participants who choose to withdraw from the study the opportunity to share their views and reasons for withdrawal in a post-study interview at the time of their exit from the study. Participants will be reassured that this is voluntary, and they need have no further contact with the research team. Discontinuing OTRTR therapy will not exclude participants from the study. Participants who wish to discontinue OTRTR will be offered the opportunity to participate in the regular assessment points should they wish. Participants who withdraw from the research study will be able to continue (if in OTRTR condition) or commence (if in TAU condition) OTRTR in the context of their usual care and treatment. Measures will be clearly prioritized so that in all assessment batteries primary measures will be administered prior to secondary minimizing the loss of primary data should participants withdraw during an assessment session. The assessment battery may be completed over two sessions to avoid participant testing fatigue.

### Protocol amendments

Any modifications to the protocol that may impact on the conduct of the study will require a formal amendment to the protocol. Such amendments will be agreed upon by the trial research team, and approved by NHS Scotland Research Ethics Committee prior to implementation, and notified to the local research committees of participating organizations in accordance with local regulations. Minor corrections and/or clarifications to the protocol, including administrative changes that have no effect on the way the study is to be conducted, will be agreed upon by the trial research team and documented. NHS Scotland Research Ethics may be notified at the discretion of the trial research team. The ISRCTN trial registration will also be updated to reflect any protocol amendments made.

### Data management

All research-generated data will be entered into password-protected electronic spreadsheets and stored separately from any participant identifying information. Participants will be assigned unique study IDs. A master list matching participant names and study IDs will be kept in a password-protected excel document and stored on a secure NHS drive. Only members of the trial research team will have access to the master list. Original forms (e.g., questionnaires) will be retained for a period of 3 years after completion of the study, stored securely on NHS Scotland premises. Anonymized data files may be retained indefinitely by the research team. To ensure accurate data collection, the optional post-study interviews will be audio recorded and transcribed for qualitative analysis. Audio data will be collected using an encrypted password-protected audio recorder and uploaded to a secure NHS drive. Data entry, preparation, and analysis will be carried out using a bespoke standardized operating procedure to ensure data integrity.

### Statistical analysis

#### Feasibility and acceptability outcomes

Descriptive statistics will be reported for the key outcomes on the feasibility data (including mean averages, standard deviations, and ranges where appropriate). The number of patients who were eligible, recruitment into the study, and attrition rates according to each intervention arm will be considered using descriptive statistics and reported using the CONSORT (Consolidated Standards of Reporting Trials) flow chart (Fig. 1). Interviews will be analyzed using thematic analysis [34] to identify emerging themes within the data.

#### Clinical outcomes

This study will not be sufficiently powered to determine treatment efficacy. Evidence of change on clinical outcomes will be considered with a focus on effect sizes and reliable change indices over null hypothesis significance testing. Effect size estimates (Cohens’ *d*) will inform sample size estimates and inform decisions regarding the most appropriate outcome measures for use in a future phase III trial of OTRTR. A modified intention to treat (ITT) analysis will be used to analyze change on the outcome measures and derive the effect size estimates. Therapeutic effect sizes (with 95% confidence intervals) will be derived using linear regression models comparing T1 scores for the two treatment arms, with baseline (T0) scores included as covariates. These analyses will be repeated using between-group comparison of means at T2 for estimated maintenance of treatment effects.

Similarly, effect sizes for change in institutional violence/aggression will be derived. The mean number of incidents of violence/aggression (recorded by the institution) measured at post treatment (the mean number of incidents for duration of study treatment phase, up to T1) will be compared, controlling for baseline differences (the mean number of incidents for the 3-months preceding treatment, up to T0). This analysis will be repeated using between-group comparison of mean incidents during follow-up period (record from T1 to T2) for estimated maintenance of treatment effect.

Changes in institutional privileges (e.g., increased grounds access, unsupervised phone calls, and patient outings) will be recorded for participants in both groups throughout the study treatment phase (T0 to T1) and follow-up period (T1 to T2). To the researchers’ knowledge, this type of outcome has not been previously studied in treatment evaluations in forensic settings. This data will be examined primarily to identify the types of changes that occur and will be used to inform secondary outcome measure selection and refinement in a future definitive RCT. Descriptive statistics will be calculated and examined.

An exploratory analysis will also estimate the individual-level effects on the two primary clinical outcome measures (Birchwood Insight Scale and the Coping Styles Questionnaire). This analysis will use the weekly BIS and CSQ scores collected during the study treatment phase and analyze change using multilevel modeling (also referred to as linear mixed effects modeling). The multilevel approach is advantageous as it accounts for dependencies among observations (repeated assessments) nested within individuals and is flexible in the presence of missing data. The analysis will reveal individual trajectories (e.g., a timecourse of therapeutic effect) and variability throughout the treatment period. The model will include two levels: participants and time (repeated observations), where time is nested within each participant. Level of treatment need (assessed at baseline) will be an additional model parameter. As for the between-group analyses which aim to estimate treatment effects, this individual-level treatment effects analysis will not be sufficiently powered for definitive hypothesis testing and results will be interpreted with caution.

To our knowledge, there is no recommended Minimal Clinically Important Difference (MCID) for this study’s primary outcome measures in forensic psychiatric populations: the Birchwood Insight Scale and the Coping Styles Questionnaire. Results from this study will be used to better understand what an appropriate MCID would be for this measure in this population. We will adopt a distribution-based approach, which compares change on the outcome measure to an index of the measure’s variability [35, 36]. This approach assumes that the outcome measure change score equivalent to a small effect size (e.g., Cohen’s *d* = 0.2) would be the MCID. The MCID will be calculated multiplying 0.2 by the standard deviation of the outcome measure across the feasibility study’s entire sample at baseline.

When data is missing, we will record reasons why missing data occurred, which will help to formulate assumptions about the missing data (e.g., missing at random) for the purpose of analysis. We will use a model-based multiple imputation method to address any missing outcome, though the exact method will be selected after a survey of the pattern of missing data.

Using the standardized recording forms completed by the therapy facilitators, each therapy session will be retrospectively coded as focusing primarily on either (1) building insight or knowledge of mental health or (2) developing coping skills. In a separate model restricted to only OTRTR condition participants, therapy session focus (measured using the standardized recording forms completed by the OTRTR facilitators) will be further included as an additional predictor of change on the clinical outcome measures.

### Data monitoring

Data and safety monitoring issues will be reviewed on a quarterly basis by the trial research team comprised of LT, SOR, and LGM. Urgent issues will be addressed as soon as possible via phone, email, or regular project meetings that include the trial research team.

A separate formal data monitoring committee will not be convened for this study due to limited resources and the feasibility nature of this study.

### Determining progression to a full trial

There are specified criteria of success in relation to participant consent (refusal < 37%), recruitment (recruit at least 40 participants) and retention (attrition < 20% at T1). Should these criteria not be met, we would not progress to a full trial using the same design. Further feasibility outcomes have been identified as critical for the decision to pursue a future RCT of OTRTR at all. For example, if this study finds significant problems with the application of the randomization procedure (e.g., non-adherence at the participating sites), this would indicate a randomized design comparing OTRTR to any comparison treatment is not feasible and should not be pursued in this treatment setting. However, most other potential feasibility or acceptability issues found by this study could be addressed by modifying the relevant procedures in a larger RCT. If significant modifications were indicated, we would consider the need for a second feasibility trial or an embedded pilot within a full-scale RCT. Effect size estimates from this feasibility study may inform the power calculation of a future RCT only if there have been minor changes to the study design between the two studies. In this case, when calculating the necessary sample size of a future trial, we would consider both the effect sizes observed in the feasibility study (using lower bound 95% confidence interval) and the minimum clinically important difference in the primary outcomes.

### Dissemination

Trial results will be communicated in conference presentations, peer-reviewed journal publications and LGM’s PhD dissertation. Consistent with the University of Edinburgh Open Access policy, findings will either be published in Open Access journals or a copy of the publication will be deposited in an Open Access Repository. Results will be disseminated in manner that maintains the confidentiality of individual participants. A summary of the results will be provided to those participants who express an interest.

## Discussion

Forensic mental health patients are often complex individuals with significant treatment needs relating to their mental health and risk. Psychological care addressing these needs must be carefully coordinated and sequenced often over a period of several years. The OTRTR psychological intervention program was developed in Scotland as an initial treatment program for patients near the start of their recovery journey with forensic mental health services. A full evaluation of OTRTR’s effectiveness is necessary to inform evidence-based clinical decision making. UK Medical Research Council guidance [37, 38] emphasizes the need to thoroughly test the feasibility and acceptability of key trial procedures prior to commencing a definitive effectiveness trial.

This study is not without its limitations. The nature of the comparison condition can greatly affect RCT outcomes and deserves substantial consideration in the design phase [39]. While a study-defined comparison condition was initially preferred by the researchers, this raised ethical and organizational concerns and we ultimately made a pragmatic decision to utilize a routine care comparison condition. Details of the care accessed by study participants will be recorded to enable a full description of what routine care entailed.

As in many evaluation studies of psychological therapies, there is a difficult balance of protocol fidelity and ensuring the treatment is responsive to participants’ needs. The multi-site nature of this evaluation increases the likelihood of variation in treatment content delivered throughout the study. However, we intend to statistically model the effects of this variation in a novel exploratory analysis using multilevel modeling. We anticipate that this method will be useful in allowing a degree of treatment flexibility within a rigorous design such as the RCT. Moreover, the ability to statistically model changes in key clinical outcomes to particular elements of the therapy protocol will add helpful insights and increase the generalizability of any OTRTR clinical effects observed.

## Abbreviations

A&R: Awareness and Recovery
AE: Adverse event
BIS: Birchwood Insight Scale
BPRS: Brief Psychiatric Rating Scale
CONSORT: Consolidated Standards of Reporting Trials
CORE-OM: Clinical Outcomes in Routine Evaluation – Outcome Measure
CSQ: Coping Styles Questionnaire
ILM: Intensive longitudinal methods
ITT: Intention to treat
LAY: Looking After Yourself
MRC: Medical Research Council
NHS: National Health Service
OTRTR: On the Road to Recovery
QPR: Questionnaire on the Process of Recovery
RCT: Randomized controlled trial
REC: Research ethics committee
RMO: Responsible Medical Officer
RSE: Rosenberg Self-Esteem Scale
SAE: Serious adverse event
TAU: Treatment as usual
